# Supplementary material for: Dosimetric outcomes of preoperative treatment planning with intraoperative optimization using stranded seeds in prostate brachytherapy
Source: PLoS One. 2022 Mar 30;17(3):e0265143. doi: 10.1371/journal.pone.0265143 (PMC8967021; doi:10.1371/journal.pone.0265143)
Supplement: S1 Fig — (DOCX) [file pone.0265143.s001.docx]

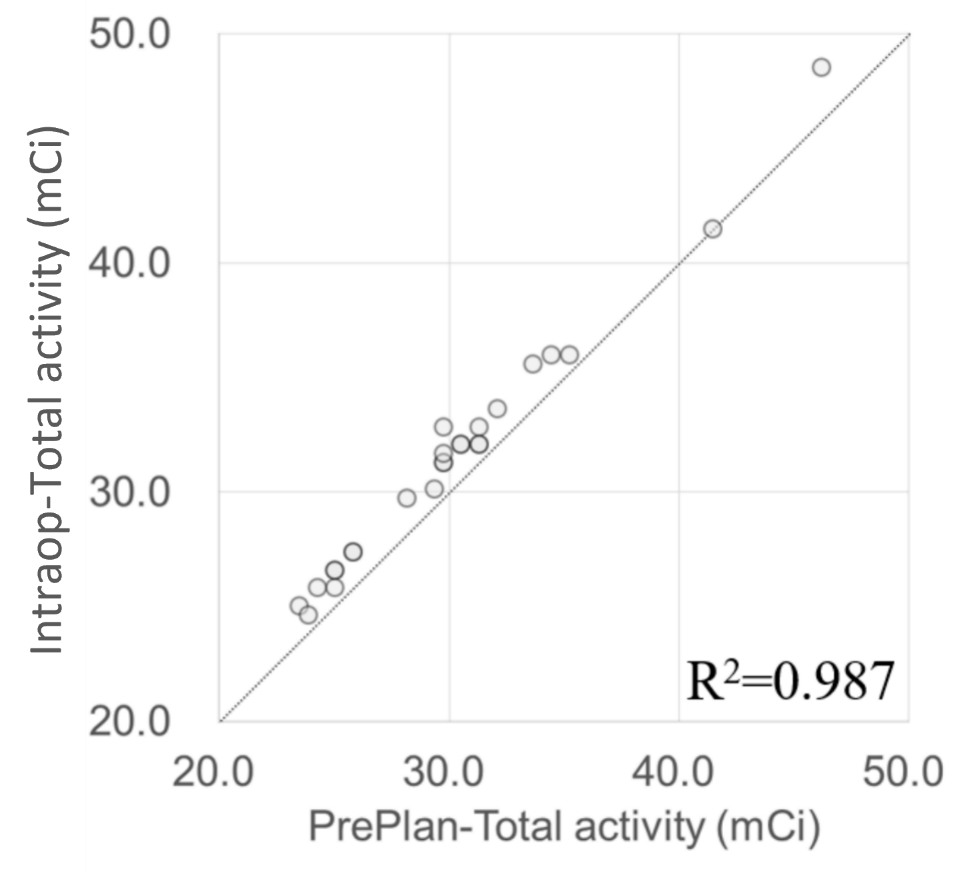


Supplementary Figure S1. Linear correlation of total activity between the preoperative and intraoperative plans in cases where additional radioactive seeds were inserted.
