# Supplementary material for: Dosimetric outcomes of preoperative treatment planning with intraoperative optimization using stranded seeds in prostate brachytherapy
Source: PLoS One. 2022 Mar 30;17(3):e0265143. doi: 10.1371/journal.pone.0265143 (PMC8967021; doi:10.1371/journal.pone.0265143)
Supplement: S2 Fig — A few seeds are located within the emerald-colored shade that indicates the planning target volume, constructed by adding a uniform margin around the red-colored prostate. (DOCX) [file pone.0265143.s002.docx]

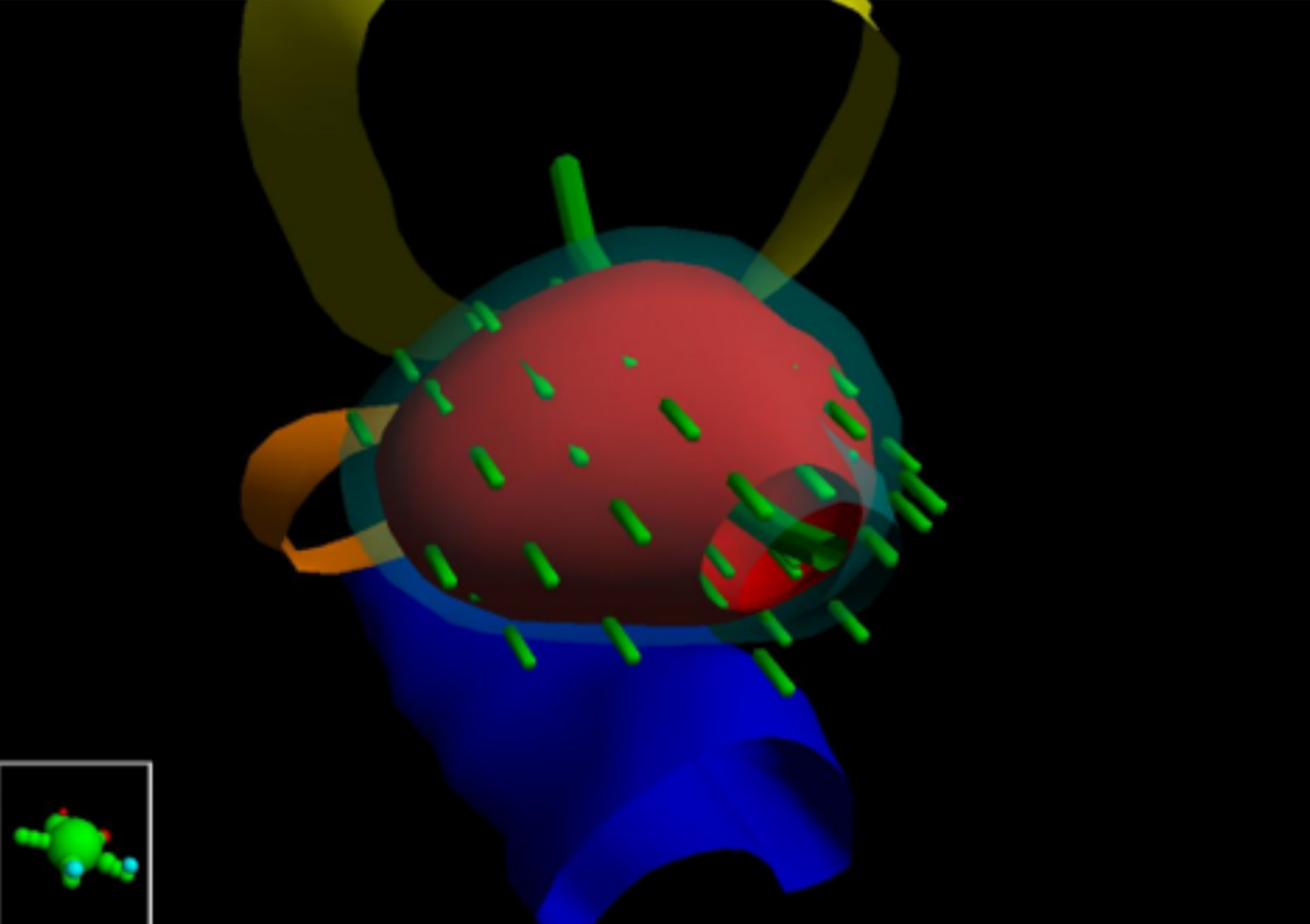


Supplementary Figure S2. Three-dimensional display of seed distribution relevant to the prostate and adjacent organs. A few seeds are located within the emerald-colored shade that indicates the planning target volume, constructed by adding a uniform margin around the red-colored prostate.
