# Supplementary material for: Dosimetric outcomes of preoperative treatment planning with intraoperative optimization using stranded seeds in prostate brachytherapy
Source: PLoS One. 2022 Mar 30;17(3):e0265143. doi: 10.1371/journal.pone.0265143 (PMC8967021; doi:10.1371/journal.pone.0265143)
Supplement: Attachment — Submitted filename: Response to reviewers.docx [file pone.0265143.s003.docx]

Reviewer #1

General Comments:

The authors reported an Ultrasound-guided transperineal prostate permanent seed implant LDR monotherapy workflow and the retrospective dosimetric analysis of 100 patient cases undergoing this procedure. Upon reviewing this manuscript, several major flaws are noted, including in presentation and in analysis. I did not find this of adequate quality for publication in its current state.

Major Comments:

1. First and foremost, it is very questionable whether this is "adaptive" planning. Adaptation generally means an optimal plan already exists based on a snapshot at a certain time point (typically sim), and adjustments are made based on anatomical or setup change at time of dose delivery. As I understand, this workflow intentionally uses/encourages a suboptimal (cold) plan/implantation to achieve urethral sparing, and then make up for the coverage intraoperatively with the spare needles (Page 7, Line 125). It is noted that Figure 4B/C shows consistently cold plans "before adaptation". This is just intraoperative optimization with Ultrasound guidance, not "adaptive", because the base plan is intentionally suboptimal to begin with.

Please clarify the workflow, or remove reference to "adaptive/adaptation" in the title and main text.

2. For the reason stated above, Figure 4B/C is very misleading. The before-after isn't really improvement but just fixing what's purposefully done wrong.

Authors’ response: Thank you for your comment. Given that comment 1 and 2 are based on the same sentiment, we would like to respond to them in a combined manner. LDR brachytherapy planning methods could roughly be classified into two categories: intraoperative real-time planning with simultaneous implantation and pre-planning with implantation as planned. Even though preoperative planning has the disadvantage of the inconvenience of an additional outpatient clinic visit for simulation, it has a clear advantage of shortening the actual procedure time under general anesthesia. In contrast, we could devote as much time as required for the most optimal construction of the plan. Moreover, intraoperative planning was impossible for us, considering that we used stranded seeds that must be customized before the procedure according to the preoperative plan. Even though the optimal implantation was not achieved in 20 cases out of 100, it was never done on purpose in the planning stage but rather accidentally during implantation. Fortunately, with the advance of the treatment planning system, a hybrid approach of preoperative planning combined with intraoperative optimization is now available, as reported by Kudchadker et al. This hybrid approach is quite important, as various events, such as human error by the practitioner and/or a slight movement of organs according to the pressure applied, could occur during the procedure. Moreover, unofficial analysis of our current data with more patients has so far showed that the ratio of optimized cases has been decreasing as more and more cases accumulate. Initially, honestly, even we were not so sure about the safety and possible hazardous effects on patients during the procedure. These hesitancies sometimes resulted in the formation of cold spots, not only around the urethra but also elsewhere, as we tried to slightly adjust the implantation during the operation to lower the dose to critical areas. Furthermore, please note that by using stranded seeds, one needle insertion leads to implantation of multiple seeds simultaneously, and could result in a somewhat unexpected systemic dose variation. Given all these considerations, we would like you to understand that we never intended to create a ‘suboptimal’ plan. We used the term ‘adaptive implantation’ in order to emphasize the effort to achieve specified dosimetric goals by improvising in some not optimally ‘implanted’ cases. This not only includes the dose to the prostate but to OARs. For instance, ‘slight’ adjustment of seed locations compared to the original plan when excessive dose to a specific OAR is anticipated. However, we do admit that this term, adaptive planning, could be misleading, as it is conventionally used in EBRT to indicate a modified plan reflecting the substantial change of tumor or normal organs. Moreover, as our current approach was referred to as ‘optimization’ by others, we have revised our title, manuscript, and Figure 1 as follows. We also would like to remove the term ‘urethral sparing’ as it could wrongly suggest that we intentionally made cold spots around the urethra.

Original: Title

Dosimetric outcomes of intraoperative optimization with adaptive implantation using stranded seeds for low-dose-rate prostate brachytherapy

Revised:

Dosimetric outcomes of preoperative treatment planning with intraoperative optimization using stranded seeds in prostate brachytherapy

Original: Page 4, line 62

Various intraoperative circumstances may prevent the implantation of seeds as planned; therefore, intraoperative dose monitoring and real-time adaptive planning are essential for quality-controlled implantation.

Revised:

Various intraoperative circumstances may prevent the implantation of seeds as planned; therefore, intraoperative dose monitoring and real-time optimization are essential for quality-controlled implantation.

Original: Page 4, line 64

Given that BT requires appropriate technical procedures, we evaluated those in detail with a description in intraoperative adaptation and MOSFET utilization.

Revised:

Given that BT requires appropriate technical procedures, we evaluated those in detail with a description in intraoperative optimization and MOSFET utilization.

Original: Page 4, line 66

Furthermore, in this study, we reported the dosimetric outcomes of LDR BT with adaptive implantation and the results of the postoperative evaluation on day 0 and day 30.

Revised:

Furthermore, in this study, we reported the dosimetric outcomes of LDR BT with optimized implantation and the results of the postoperative evaluation on day 0 and day 30.

Original: Page 6, line 103

Surgical procedures and intraoperative adaptation

Revised:

Surgical procedures and intraoperative optimization

Original: Page 7, line 121

When the delivered seed positions or dose distribution did not match those in the preoperative plan, the implant was optimized in the operating room, and additional seeds were inserted to ensure complete target coverage, while also ensuring that the prostate V100% was > 95%, the rectum V100% was < 1 cc, and the urethra V200 Gy was 0 cc. Therefore, a total of four spare needles, two needles with two extra seeds and two needles with three extra seeds, were prepared for each patient.

Revised:

When the dose distribution did not match that of the preoperative plan due to various intraoperative factors, the implant was optimized in the operating room, and additional seeds were inserted to ensure sufficient target coverage, while also ensuring that the prostate V100% was > 95%, the rectum V100% was < 1 cc, and the urethra V200 Gy was 0 cc. Therefore, a total of four spare needles, two needles with two extra seeds and two needles with three extra seeds, were prepared in case of target underdose for each patient.

Original: Page 9, line 180

The linear correlation of total activity between the preoperative plan and intraoperative optimization in patients who required additional radioactive seeds inserted for adaptive implantation are presented in Supplementary Figure S1.

Revised:

The linear correlation of total activity between the preoperative plan and intraoperative optimization in patients who required additional radioactive seeds inserted are presented in Supplementary Figure S1.

Original: Page 10, line 182

At the end of the implantation, if the intraoperative dose optimization result showed that the V100% of the prostate was < 95% or D90% was < 140 Gy, this would be considered insufficient target coverage, and adaptive implantation would be performed by inserting additional seeds.

Revised:

At the end of the implantation, if the intraoperative dose optimization result showed that the V100% of the prostate was < 95% or D90% was < 140 Gy, this would be considered insufficient target coverage, and additional seeds were inserted for compensation.

Original: Page 10, line 185

Adaptive implantation was performed in 20 patients by inserting 2–5 additional stranded seeds.

Revised:

In 20 patients, 2–5 additional stranded seeds were inserted.

Original: Page 10, line 194

Figure panels 4B and 4C present the reduction of the difference between the dose distribution in the preoperative simulation and the intraoperative dose calculation by adaptive implantation.

Revised:

Figure panels 5B and 5C present the reduction of the difference between the dose distribution in the preoperative simulation and the intraoperative dose calculation by additional compensatory implantation.

Original: Page 10, line 196

These figure panels show that adaptive implantation through intraoperative dynamic optimization can improve the dosimetric outcomes of LDR BT.

Revised:

These figure panels show that intraoperative dynamic optimization can improve the dosimetric outcomes of LDR BT.

Original: Page 17, line 362

Linear correlation of total activity between the preoperative and intraoperative plans in cases where additional radioactive seeds were inserted for adaptive implantation

Revised:

Linear correlation of total activity between the preoperative and intraoperative plans in cases where additional radioactive seeds were inserted.

Original: Page 23, line 555

After seed implantation, TRUS-based dosimetry is performed. When the target coverage was not sufficient, intraoperative optimization and adaptation were conducted immediately.

Revised:

After seed implantation, TRUS-based dosimetry is performed. When the target coverage was not sufficient, intraoperative optimization was conducted immediately.

Original: Page 24, line 569

Fig. 5. (A) D90% and V145 Gy for preoperative planning, before and after intraoperative adaptation. (B) The difference between the intraoperative and preoperative plan in D90% was divided by the preoperative D90%. (C) The difference between the intraoperative and preoperative plan in V145 Gy was divided by the preoperative V145 Gy. In (B) and (C), light bars indicate the calculated value before adaptation and dark bars indicate the calculated value after adaptation.

Revised:

Fig. 5. (A) D90% and V145 Gy for preoperative planning, before and after intraoperative optimization. (B) The difference between the intraoperative and preoperative plan in D90% was divided by the preoperative D90%. (C) The difference between the intraoperative and preoperative plan in V145 Gy was divided by the preoperative V145 Gy. In (B) and (C), light bars indicate the calculated value before optimization and dark bars indicate the calculated value after optimization.

Original: Page 24, line 586

Scatter plots showing (a) the urethra V200 Gy and (b) rectum V100%. The values suggest that the doses to the urethra and rectum were maintained at around or lower than the threshold after implantation. In both plots, light dots indicate the values on day 0 and dark dots indicate the values on day 30. Demonstrably, highly controlled implantation was achieved in all cases to spare the organs at risk.

Revised:

Scatter plots showing (a) the urethra V200 Gy and (b) rectum V100%. The values suggest that the doses to the urethra and rectum were maintained at around or lower than the threshold after implantation. In both plots, light dots indicate the values on day 0 and dark dots indicate the values on day 30. Demonstrably, highly controlled implantation was achieved in all cases without excessive dose to the organs at risk.

Original: Figure 1

Revised:

3. V100% or V200% are reported in % rather than cc. How is this normalized in post-op, especially in case of prostate volume change. There is no mentioning of this detail.

Authors’ response: Thank you for your comment. However, given the variation in the prostate volume across patients, we think it is more likely to be misleading if we provide V100% or V200% in cc. For instance, if two patients had identical V100% in terms of cc, it could be either proper, insufficient, or excessive dose coverage to the prostate, depending on the patient’s baseline prostate volume. Probably, this could be the reason that the majority of studies we referenced reported dose-volume parameters in percentage rather than volume. We also would like to clarify that we could not observe substantial prostate volume change in postoperative CT in our cohort. Hypothetically, this could be the result of stranded seed implantation with a lesser number of needle insertions than multiple individual seed implantation, which requires as many needle insertions. According to your comment, we would like to add a detailed explanation regarding the postoperative dosimetry process. The revisions made in the manuscript have been provided after our response to comment #4.

4. The D90(Intraop) - D90(Preop) metric which the authors refer to throughout the manuscript seems flawed, without an analysis comparing the individual seed positions and those in the preop plan.

Authors’ response: Thank you for your comment. As D90% is one of the most widely utilized dosimetric parameters for defining the quality of prostate brachytherapy, along with V100%, in many other studies [1, 2], we think it is plausible to use the D90% metric for evaluating the dosimetric quality of low-dose rate prostate brachytherapy. Further, regarding individual seed positions, please understand that we use a brachytherapy needle insertion template with indicative coordinates. This template is correlated to the TRUS system, enabling the reproduction of seed position to be identical to that seen in the preoperative simulation. Even though we mentioned possible flaws during implantation in our response to your comments 1 and 2, the difference in individual seed positions was within the boundary of acceptance. Please note that, we therefore removed the phrase ‘the delivered seed positions’ from the manuscript (see our revised manuscript according to comment 1&2) to avoid confusion. The focus of our analysis was the importance of the dose to the given proportion of the total volume after completion of the procedure, rather than a separate analysis regarding the location of each seed. According to your comments 3 and 4, we would like to revise our manuscript as follows.

Original: Page 7, Line 128

An immediate postoperative computed tomography (CT) scan in the supine position was also obtained to calculate the detailed dose distribution according to the actual seed implantation.

Revised:

Postoperative dosimetry

CT-based postoperative dosimetry was evaluated using CT images acquired for both Day 0 (immediately after the procedure) and Day 30. All postoperative CT images were obtained with a slice thickness of 1 mm for appropriate visualization of the implanted seeds. The detailed process was as follows. First, on the CT image, VariSeed software version 8.0.1. automatically localized the implanted seeds by thresholding CT numbers, and the physician confirmed the property of identified seeds locations. Second, all of the contours generated by TRUS, except the rectum, were rigidly aligned to the postoperative CT images. The rectum was manually contoured regarding its substantial geometrical change in the CT image compared to the TRUS image. Finally, the radiation dose to the prostate and OARs was calculated with the localized brachytherapy seeds. All of the computational dosimetries, including preoperative, intraoperative, and postoperative dosimetry, were performed using VariSeed 8.0.1. Specifically, radiation doses were calculated using a point source model, based on the updated American Association of Physicists in Medicine (AAPM) TG-43 formalism. The air-kerma strength of the I-125 source was set to 0.391 mCi.

5. The concept of intraoperative optimization isn't new. Overall, this work doesn't seem to add much beyond what's reported in Kudchadker et al. doi: 496 10.1016/j.meddos.2012.03.001.

Authors’ response: Thank you for your comment. Your comment somehow exposed one of the few limitations of our current study. As low-dose-rate brachytherapy nowadays has become one of the conventional treatment modalities in early-stage prostate cancer, especially in the western hemisphere, this study may not contribute significantly towards innovation in the field. However, we tried to add some new features to this saturated area by reporting the sophistication of the methodology, with the results of a pilot study using MOSFET. Furthermore, we would like to report the practical outcomes of intraoperative optimization at our institution. Even though this study is not conceptually revolutionary, we think that this kind of descriptive study could have its own advantages, providing a detailed technical guideline to those who would like to launch a low-dose-rate brachytherapy system at their institution. As PLOS one declares its publication standard based on scientific rigor rather than novelty, we think that this journal would be an appropriate platform to publish our data.

6. The use of intraoperative MOSFET dosimetry is interesting --- is this used to guide optimization?

Authors’ response: Thank you for your comment. We acquired 2-minute in-vivo measurements during the operation according to the physician’s requests, and 2-minute and 10-minute in-vivo dosimetry to acquire life-time radiation doses after completion of the whole operation. Recently, intraoperative MOSFET dosimetry has been actively employed as a touchstone in our practice. For instance, assume a situation where the urethral dose is much higher than expected, based on 2-minute intraoperative MOSFET measurements; then, the remaining seeds could be located a little bit further away from the urethra than proposed in the initial preoperative plan. Even though this kind of event occurs very rarely, we are currently using intraoperative MOSFET as one of the tools for optimization. Within this study cohort, however, it was used at the pilot test phase to examine its plausibility, rather than using it for optimization guidance. Therefore, we think the most accurate response to your question is that we use intraoperative MOSFET dosimetry as optimization guidance ‘currently’, but did not do so in the period of this study. For providing a better understanding to the readers, we would like to add more details regarding the process of MOSFET dosimetry in the manuscript.

Original: Page 6, line 110

The schematic description of the relative location of MOSFET, TRUS, and inserted seeds is shown in Figure 2.

Revised:

The schematic description of the relative location of MOSFET, TRUS, and inserted seeds is shown in Figure 2. Two-minute in-vivo MOSFET measurements were performed intraoperatively, and 2-minute and 10-minute measurements were acquired at the end of the surgical procedure. The electrical bias measured by the MOSFET detectors was converted using a vendor-provided calibration factor (15.2 mV/cGy). These measured doses were converted to the life-time radiation doses, which the patient would receive during the whole decaying period of the implanted seeds. The maximum value of the 10-minute measurement results was compared to the maximum urethral dose in the preoperative plan, further assuring the dosimetric quality of the procedure.

Instead of just reporting the max and mean in-vivo reading (Page 9, Line 167), I recommend adding a detailed plot combing the in-vivo data and TPS dose of the urethra.

Authors’ response: Thank you for the comment. Regarding your suggestion, we hereby provide a plot (PLOT 1) displaying the in-vivo maximum urethral dose of 50 patients. However, please understand that this procedure, as mentioned above, was one of our initial attempts to assure that the urethra had not been exposed to excessive radiation. This was a rather experimental approach for us back then, not knowing how the results of this in-vivo measurement would unfold. As we mentioned in the manuscript, this MOSFET measurement was done in five spots within the foley catheter; however, its exact position in accordance with the TPS was not accurate. This means that the maximum dose measured by MOSFET could not be matched to maximum urethral dose measured by TPS. Even though it would be great if we could provide a point-to-point comparison of in-vivo measurement with its corresponding urethral dose in the TPS, this was not possible in our current study. As we are gathering more data from in-vivo measurements recently, with more refined treatment techniques and process, research efforts focusing solely on the physical property of MOSFET are underway.

Another thing to consider is that the urethral maximum dose does not have much ‘clinical’ significance unless it exceeds 200 Gy in a practical setting. Treatment planning aimed to keep the dose to the prostate and other OARs to be within the predefined range, rather than achieve a specific value. That is why we provided the median in-vivo measured maximum dose and range to show that almost all patients had a urethral maximum dose less than 200 Gy. In the current study, only two patients recorded a urethral maximum dose greater than 200 Gy, among which, one case was likely the result of a technical error.

PLOT 1.

6. The workflow needs to be presented much more clearly. Specifically:

a. There is no pre-planning image/dose in the main text at all. Supplementary Figure S2 should be prominently placed in the main text.

Authors’ response: Thank you for the comment. We would like to include supplementary figure S2 in the main text, as you recommended. As supplementary figure S2 becomes Figure 4, the sequence of other figures would change as well. However, we thought that inserting a preoperative plan into the main text could be redundant, as it would have no significance other than just adding plain images. Please understand that the preoperative plan itself in this study would have had no substantial difference from other exemplary images of a brachytherapy plan available elsewhere.

Original: Page 10, line 191

Supplementary Figure S2 shows an example of additional seed implantation in the central portion of the prostate while maintaining the dose constraints for OARs with improved prostate dose coverage.

Revised:

Figure 4 shows an example of additional seed implantation in the central portion of the prostate while maintaining the dose constraints for OARs with improved prostate dose coverage.

b. Page 7 Line 113 says intraop images were compared with sim. How? On the TPS?

Authors’ response: Thank you for the comment. While TPS itself could provide a real-time comparison between intraoperative images and preoperative images, along with contours drawn in advance, we have multiple other options for comparing intraoperative images with preoperatively acquired images. Given that we have many licenses for the TPS, we could simultaneously display as many images as required, on demand. In the operating room, during implantation, intraoperative TRUS images and preoperative images were shown side by side on large monitors attached to the ceiling by mechanical arms. We also had separate laptops, mainly used by assisting physicians or physicists, that could display whatever images we wanted. According to your comment, we would like to revise the manuscript as follows.

Original: Page 7, line 113

These intraoperative images were then compared with corresponding images obtained during preoperative simulation to determine the necessity of intraoperative improvisation of needle insertion.

Revised:

These intraoperative images were then compared with corresponding images obtained during preoperative simulation on monitors installed in the operating room to determine the necessity of intraoperative improvisation of needle insertion.

c. Page 7 Line 115 says the dose to OARs is evaluated intraoperatively. Is this simply overlay the dose distribution from pre-op plan onto current image? Any registration performed?

Authors’ response: Thank you for the comment. TPS provides real-time dose calculation according to the position of implanted seeds. As shown in supplementary figure S2 (now figure 4), orange circles that were marked by a physician indicate the position of implanted seeds. TPS automatically calculated the dose distribution regarding this position of seeds once they were designated. Therefore, it is not just a simple overlay or registration of preoperative plan onto current intraoperative images. Rather, in accordance with the answer to comment 6b, it was a one-on-one comparison between preoperative plan and intraoperative dose distribution constructed instantaneously by TPS. We would like to revise our manuscript as follows. Explanation for small change regarding recontouring is given in the response to comment 6e.

Original: Page 7, line 115

The estimated radiation dose to other OARs was contemporaneously analyzed based on the TRUS images acquired during the operation. Although the target and OARs remained consistent throughout the period between the simulation and operation, contours had to be delineated again in a few cases. Intraoperative dose calculation was also performed with the software used for preoperative simulation, and the results of these analyses were compared with the preoperative plan to determine whether the actual implanted seeds could fulfill the abovementioned dose parameters.

Revised:

The prostate and OARs remained consistent throughout the period between the simulation and operation; therefore, contours had to be delineated again only in rare cases. The real-time estimated radiation dose to the prostate and other OARs was contemporaneously calculated by VariSeed software version 8.0.1., based on the TRUS images and seed locations acquired during the operation. Results of these analyses were compared with the preoperative plan to determine whether the actual implanted seeds could ultimately fulfill the abovementioned dose parameters.

Original: Figure caption of Fig. 4. (former S2)

An exemplary case of cold-spot reduction around the urethra and improvement of dosimetric parameters. With real-time intraoperative optimization, both better dose coverage and maintained dose constraints were possible.

Revised:

An exemplary case of cold-spot reduction around the urethra and improvement of dosimetric parameters. Red lines are isodose lines for prostate V100%, orange circles indicate the actual implanted seed locations, and real-time dose distribution was calculated instantaneously according to these locations. With real-time intraoperative optimization, both better dose coverage and maintained dose constraints were possible.

d. What is the PTV? Any margins? From the manuscript, it seems to imply the prostate gland itself is the planning volume with no margins. Please clarify.

Authors’ response: Thank you for the comment. PTV was defined as the prostate plus a 3-mm margin in the anterior, left, and right directions. Given that the rectum is located just behind the prostate, and TRUS locates within the rectum, we did not apply any margin posteriorly. As you pointed out, the dose constraint for the prostate mentioned in this study was based on the prostate gland itself, with no margin. We routinely construct PTV for sake of reference rather than applying dosimetric significance to it. Further, as we frequently implant a few seeds around the prostate, as shown in supplementary figure S3 (now supplementary figure S2), PTV serves as an ultimate outer margin for seeds implantation. However, we should admit that this could cause confusion, as PTV usually reflects the setup margin in EBRT planning. To avoid further misunderstanding, we would like to remove the PTV volume row from the table, as PTV was not an object for dose-volume analysis in the current study.

Original:

| Table 2. Dosimetric characteristics |  |
| --- | --- |
| Dosimetric characteristics | Median (range) |
| No. of needles implanted | 20 (14-33) |
| No. of seeds used | 76 (52-102) |
| *Preoperative dosimetry* |  |
| Prostate volume (cc) | 28.68 (10.39-48.01) |
| PTV^*^ (cc) | 37.72 (15.69-65.90) |
| Prostate V_100%_ (%) | 95.72 (91.33-98.48) |
| Prostate V_200%_ (%) | 18.63 (9.39-34.73) |
| Prostate D_90%_ (Gy) | 159.64 (148.26-172.06) |
| Rectum V_100%_ (cc) | 0.16 (0.00-0.67) |
| Urethra V_200 Gy_ (cc) | 0.00 (0.00-0.00) |
| *Postoperative dosimetry - Day 0* |  |
| Prostate V_100%_ (%) | 92.28 (76.63-97.81) |
| Prostate V_200%_ (%) | 18.63 (9.39-34.73) |
| Prostate D_90%_ (Gy) | 150.88 (131.02-174.89) |
| Rectum V_100%_ (cc) | 0.11 (0.00-1.90) |
| Urethra V_200 Gy_ (cc) | 0.00 (0.00-0.03) |
| *Postoperative dosimetry - Day 30* |  |
| Prostate V_100%_ (%) | 92.23 (80.50-100.00) |
| Prostate V_200%_ (%) | 25.02 (10.77-81.99) |
| Prostate D_90%_ (Gy) | 151.46 (126.00-181.35) |
| Rectum V_100%_ (cc) | 0.22 (0.00-1.70) |
| Urethra V_200 Gy_ (cc) | 0.00 (0.00-0.26) |
| *Planning target volume |  |

Revised:

| Table 2. Dosimetric characteristics |  |
| --- | --- |
| Dosimetric characteristics | Median (range) |
| No. of needles implanted | 20 (14-33) |
| No. of seeds used | 76 (52-102) |
| *Preoperative dosimetry* |  |
| Prostate volume (cc) | 28.68 (10.39-48.01) |
| Prostate V_100%_ (%) | 95.72 (91.33-98.48) |
| Prostate V_200%_ (%) | 18.63 (9.39-34.73) |
| Prostate D_90%_ (Gy) | 159.64 (148.26-172.06) |
| Rectum V_100%_ (cc) | 0.16 (0.00-0.67) |
| Urethra V_200 Gy_ (cc) | 0.00 (0.00-0.00) |
| *Postoperative dosimetry - Day 0* |  |
| Prostate V_100%_ (%) | 92.28 (76.63-97.81) |
| Prostate V_200%_ (%) | 18.63 (9.39-34.73) |
| Prostate D_90%_ (Gy) | 150.88 (131.02-174.89) |
| Rectum V_100%_ (cc) | 0.11 (0.00-1.90) |
| Urethra V_200 Gy_ (cc) | 0.00 (0.00-0.03) |
| *Postoperative dosimetry - Day 30* |  |
| Prostate V_100%_ (%) | 92.23 (80.50-100.00) |
| Prostate V_200%_ (%) | 25.02 (10.77-81.99) |
| Prostate D_90%_ (Gy) | 151.46 (126.00-181.35) |
| Rectum V_100%_ (cc) | 0.22 (0.00-1.70) |
| Urethra V_200 Gy_ (cc) | 0.00 (0.00-0.26) |

e. During the intra-operative procedure, are target and OAR contours re-drawn sometimes or always? The manuscript seems to indicate this is case-by-case --- what's the criteria then?

Authors’ response: Thank you for the comment. In most cases, there was no significant difference in the location and volume of prostate and other OARs. The decision to redraw contours intraoperatively in some patients was based solely on the physician's discretion, without defined criteria. For instance, if intraoperative TRUS images showed a significant transformation of the prostate, for example, even evading a range of predefined PTV, then the physician would probably have recontoured the prostate. However, this does not happen frequently, and we have not felt the need to make up specific criteria for recontouring so far. According to your comment, we would like to revise the manuscript as follows.

Original: Page 7, line 115

The estimated radiation dose to other OARs was contemporaneously analyzed based on the TRUS images acquired during the operation. Although the target and OARs remained consistent throughout the period between the simulation and operation, contours had to be delineated again in a few cases.

Revised:

The prostate and OARs remained consistent throughout the period between the simulation and operation; therefore, contours had to be delineated again only in rare cases.

f. Page 7 Line 127 says that kV radiograph is acquired after each "operation". Please clarify the word "operation" --- Is this after the entire OR procedure, or after each needle insertion?

Authors’ response: Thank you for the comment. The word “operation” meant the entire OR procedure. We do not take a kV radiograph after each needle insertion. We would like to revise the manuscript as follows.

Original: Page 7, line 127

A kidney, ureter, and bladder radiograph was acquired after each operation to observe the gross distribution of the implanted seeds in the prostate area.

Revised:

A kidney, ureter, and bladder radiograph was acquired after entire operation to observe the gross distribution of the implanted seeds in the prostate area.

7. First paragraph of Discussion is over-editorializing, comparing LDR and EBRT but ignoring HDR. I recommend removing this paragraph in its entirety as it's unrelated to the results of this work.

Authors’ response: Thank you for the comment. We definitely had no intention of ignoring HDR. However, contemplating your comment, we understand that this paragraph was too lengthy, without much essence in it. Therefore, to make the manuscript concise and engage the readers better, we would like to revise the manuscript as follows. Please understand that we retained a small portion of the paragraph, as this paragraph serves as an ‘introduction’ to the Discussion. The sequence of references has also been changed according to the removal of some references within this paragraph.

Original: LDR BT with permanent seed implantation is a safe and effective treatment modality for the management of early-stage prostate cancer. In the treatment of early-stage prostate cancer, both treatment efficacy and treatment-related toxicity are important considerations. Surgery tends to induce adverse effects, such as urinary incontinence and erectile dysfunction, in patients with prostate cancer [1, 3, 13-16]. Apart from surgery, both LDR BT and EBRT can be effectively used to manage prostate cancer. Many studies have reported that modern-era EBRT with a dose > 70 Gy leads to improved long-term outcomes compared to a radiation dose < 70 Gy [17-19]. However, further dose escalation is limited because of the increased risk of developing toxicity in adjacent OARs, such as the bladder or rectum. BT has a relative advantage in terms of dose escalation, as the physical prescription dose and biological effective dose can be up to 2–3 times greater than those in EBRT [20]. Another distinctive feature of LDR BT, unlike EBRT, is the heterogeneity of the dose distribution in the prostate and OARs. A high dose of radioactivity could be achieved around the seed implanted area, while a cold spot could exist if the seeds are not implanted properly or if seed migration occurs. Due to the precautious approach, cold spots that required additional seed implantation occasionally occurred around the urethra in our practice. It is disputable whether this property of LDR BT is beneficial in tumor control; it could be quite beneficial in lowering the dose distribution to the OARs [10, 21-24]. Considering these advantages of LDR BT over EBRT, LDR BT should be retained as an important modality in managing prostate cancer in the era of cutting-edge robotic surgery and modern EBRT.

Revised:

LDR BT with permanent seed implantation is a safe and effective treatment modality for the management of early-stage prostate cancer. In the treatment of early-stage prostate cancer, both treatment-related toxicity and treatment efficacy are important considerations. BT tends to induce fewer adverse effects, such as urinary incontinence and erectile dysfunction, compared to surgery [1, 3, 15-17]. BT also has a relative advantage in terms of dose escalation, as the physical prescription dose and biological effective dose can be up to 2–3 times greater than those in EBRT [18].

Minor Comments:

Thank you for your comments. We revised our manuscript as follows regarding your minor comments.

1. Page 4, Line 49: Remove the extra letters behind [1-3]

Original:

BT is associated with excellent cancer-related outcomes and results in remarkable improvement in quality of life, including reduction of urinary incontinence and sexual dysfunction, compared to other radical treatment strategies [1-3]stra.

Revised:

BT is associated with excellent cancer-related outcomes and results in remarkable improvement in quality of life, including reduction of urinary incontinence and sexual dysfunction, compared to other radical treatment strategies [1-3].

2. Supplementary S1. Y-axis. The shorthand for intraoperative is misspelled.

Original:

Revised:

Reviewer #2

General Comments:

This manuscript evaluated prostate LDR plan quality and reported dosimetric outcomes and early clinic outcomes. The study might be beneficial to the clinical application. However, authors should describe the LDR adaptive treatment in more details and it would be better to give some clinical suggestions at the end. Here are my comments and suggestions:

Major Comments:

1. What is the dose calculation algorithm used in this study? TG43? What source model was used: point source or line source? As this study reported plan dosimetric characteristic, details about dose calculation should also be reported.

Authors’ response: Thank you for the comment. We totally agree with you that more detailed information on the dose calculation algorithm is required. We used a point-source dose calculation model based on the updated TG-43 formalism. According to your comment, we have added the following explanations regarding postoperative dosimetry and dose calculation.

Original: Page 7, Line 128

An immediate postoperative computed tomography (CT) scan in the supine position was also obtained to calculate the detailed dose distribution according to the actual seed implantation.

Revised:

Postoperative dosimetry

CT-based postoperative dosimetry was evaluated using CT images acquired for both Day 0 (immediately after the procedure) and Day 30. All postoperative CT images were obtained with a slice thickness of 1 mm for appropriate visualization of the implanted seeds. The detailed process was as follows. First, on the CT image, VariSeed software version 8.0.1. automatically localized the implanted seeds by thresholding CT numbers, and the physician confirmed the property of identified seeds locations. Second, all of the contours generated by TRUS, except the rectum, were rigidly aligned to the postoperative CT images. The rectum was manually contoured regarding its substantial geometrical change in the CT image compared to the TRUS image. Finally, the radiation dose to the prostate and OARs was calculated with the localized brachytherapy seeds. All of the computational dosimetries, including preoperative, intraoperative, and postoperative dosimetry, were performed using VariSeed 8.0.1. Specifically, radiation doses were calculated using a point source model, based on the updated American Association of Physicists in Medicine (AAPM) TG-43 formalism. The air-kerma strength of the I-125 source was set to 0.391 mCi.

2. MR image was acquired preoperatively and stored in the MIM. Is this only used for prostate volume measurement? This is not included Figure1.

Authors’ response: Thank you for the comment. Most patients visiting our outpatient clinic usually have already undergone PSA exams and MRI scanning, and have been diagnosed with early-stage prostate cancer. Almost all patients have not made their decision when they come to the outpatient clinic, but are considering from among multiple options. They usually approach our clinic through various ways, including a referral from the urologist, prostate cancer patients’ social network, internet searching, etc. Therefore, during their first visit, the physician needs to let them know, at the very least, whether or not they are eligible candidates for brachytherapy. Given that the volume of the prostate is a very important factor in determining the eligibility, we measured the prostate volume through MR images, using the MIM software as a part of screening process. If the prostate volume is too large, then either other treatment modalities should be recommended or hormone therapy should be attempted to reduce prostate volume. It is, therefore, not a component of our brachytherapy process, but used for the sake of initial screening. According to your comment, we would like to revise the manuscript as follows.

Original: Page 5, line 85

Preoperatively, magnetic resonance (MR) images of each patient were acquired and transferred to MIM software version 6.4.6 (MIM Software Inc., Cleveland, OH, USA), and the prostate volume was measured based on these images. In patients with an initial prostate volume larger than 50 cc, neoadjuvant androgen deprivation therapy was performed before BT to reduce the volume below 50 cc.

Revised:

Preoperatively, magnetic resonance (MR) images of each patient were acquired and transferred to MIM software version 6.4.6 (MIM Software Inc., Cleveland, OH, USA), and the prostate volume was measured based on these images. These MR images were used to determine whether the patient is eligible for BT. In patients with an initial prostate volume larger than 50 cc, neoadjuvant androgen deprivation therapy was performed before BT to reduce the volume below 50 cc.

3. In this study, MR, TRUS and CT images were used. It would be better to describe the purpose of these imaging. In addition, multiple software were mentioned like MiM and Variseed. When describe the process, like in line 119, please specified which software was used in each step.

Authors’ response: In continuation of our previous response, MR images were also used for diagnosis and localization of the tumor. However, please understand that this is irrelevant to our BT procedure, but is related to general diagnosis of the prostate cancer, and therefore, we are not going to mention it in the manuscript. TRUS was used for acquiring images for preoperative planning and actual implantation. CT was the best option for postoperative dosimetry at day 0 and day 30 with implanted seeds monitoring. Please see the revisions made, as indicated in the responses to your comments #1 and #2, as we revised the manuscript, adding an explanation regarding the usage of MR & CT images. Regarding TRUS images, as these images are the main templates of our BT procedure, we revised our manuscript to allow a better understanding, as follows. Further, please note that we tried to clarify the software we used for describing the postop dosimetry process mentioned above, and revised line 119 as follows.

Original: Page 7, line 112

These intraoperative images were then compared with corresponding images obtained during preoperative simulation to determine the necessity of intraoperative improvisation of needle insertion. The estimated radiation dose to other OARs was contemporaneously analyzed based on the TRUS images acquired during the operation. Although the target and OARs remained consistent throughout the period between the simulation and operation, contours had to be delineated again in a few cases.

Revised:

These intraoperative images were then compared with corresponding images obtained during preoperative simulation on monitors installed in the operating room to determine the necessity of intraoperative improvisation of needle insertion. The prostate and OARs remained consistent throughout the period between the simulation and operation; therefore, contours had to be delineated again only in rare cases. The real-time estimated radiation dose to the prostate and other OARs was contemporaneously calculated by VariSeed software version 8.0.1. based on the TRUS images and seed locations acquired during the operation. Results of these analyses were compared with the preoperative plan to determine whether the actual implanted seeds could ultimately fulfill the abovementioned dose parameters.

Original: Page 7, line 118

Intraoperative dose calculation was also performed with the software used for preoperative simulation, and the results of these analyses were compared with the preoperative plan to determine whether the actual implanted seeds could fulfill the abovementioned dose parameters.

Revised:

Intraoperative dose calculation was also performed with VariSeed software version 8.0.1., and the results of these analyses were compared with the preoperative plan to determine whether the actual implanted seeds could fulfill the abovementioned dose parameters.

4. More details about planning should be included in the manuscript. Like what technique was used? How to do the optimization? How did the registration help the intraoperative plan?

Authors’ response: Thank you for the comment. We used a modified peripheral loading technique to avoid urethral injury as far as possible [3, 4]. Our practice consists of a two-step approach, with preoperative planning and actual implantation according to the plan, with intraoperative optimization. Basically, we do not make an intraoperative plan separately. Therefore, the strength of this two-step approach is the reduction of actual operation time under general anesthesia when compared to one-step intraoperative planning and implantation. However, intraoperative optimization was necessary from time to time, specifically in 20 patients in our cohort, because of the unpredictive nature of the actual procedure, including a slight deviation of anatomy and possible human error. As shown in the supplementary figure S2 (now figure 4), revised according to the comment from another reviewer, orange circles indicate the position of implanted seeds. TPS automatically calculated the dose distribution regarding this position of seeds once they were pointed out. Therefore, the optimization of the process consists of every modification to achieve optimal dosimetric goals based on intraoperative dose distribution constructed instantaneously by the TPS after every needle insertion. According to your comment, along with our addition of postoperative dosimetry details, we would like to modify the manuscript as follows.

Original: Page 6, line 104

Patients received seed implantation in a supine lithotomy position identical to the simulation. Stranded iodine-125 seeds (Best Medical International Inc., Springfield, VA, USA) were used in all patients.

Revised:

Patients received seed implantation through a modified peripheral loading technique to avoid urethral injury in a supine lithotomy position identical to the simulation. Stranded iodine-125 seeds (Best Medical International Inc., Springfield, VA, USA) were used in all patients.

Original: Page 10, line 191

Supplementary Figure S2 shows an example of additional seed implantation in the central portion of the prostate while maintaining the dose constraints for OARs with improved prostate dose coverage.

Revised:

Figure 4 shows an example of additional seed implantation in the central portion of the prostate while maintaining the dose constraints for OARs with improved prostate dose coverage.

Original: Page 7, line 113

These intraoperative images were then compared with corresponding images obtained during preoperative simulation to determine the necessity of intraoperative improvisation of needle insertion.

Revised:

These intraoperative images were then compared with corresponding images obtained during preoperative simulation on monitors installed in the operating room to determine the necessity of intraoperative improvisation of needle insertion.

5, MOSFET was used in this study to verify the urethral dose. How to calibrate MOSFET? As energy of seeds is low compared with external beam radiation therapy. How to get the in-vivo measurement?

Authors’ response: Thank you for your comments. The point-by-point responses to your comments are provided below.

First, regarding the MOSFET calibration, the calibration factor provided by the vendor was used (15.2 mV/cGy).

Second, the Linear 5ive Array model (TN-502LA5, Best Medical Canada, Ottawa, ON, Canada), which is in clinical use at the authors’ institute, is specifically designed for the dose measurement of LDR brachytherapy. For internal calibration, it is desirable to use a radiation source with similar energy levels to the LDR brachytherapy source (I-125). Therefore, a calibration procedure using I-125 seeds is under development as an ongoing research project.

Finally, in vivo measurement was performed by inserting a MOSFET dosimeter and a Foley catheter, as described in the manuscript. In the middle of the surgical procedure, a 2-minute measurement was made. In addition, another 10-minute measurement was made upon the completion of the procedure. These measurements were converted to the life-time doses, which the patient will receive during the whole decaying period of the implanted seeds.

In response to your comment, we would like to revise the manuscript as follows.

Original: Page 6, line 110

The schematic description of the relative location of MOSFET, TRUS, and inserted seeds is shown in Figure 2.

Revised:

The schematic description of the relative location of MOSFET, TRUS, and inserted seeds is shown in Figure 2. Two-minute in-vivo MOSFET measurements were performed intraoperatively, and 2-minute and 10-minute measurements were acquired at the end of the surgical procedure. The electrical bias measured by the MOSFET detectors was converted using a vendor-provided calibration factor (15.2 mV/cGy). These measured doses were converted to the life-time radiation doses, which the patient would receive during the whole decaying period of the implanted seeds. The maximum value of the 10-minute measurement results was compared to the maximum urethral dose in the preoperative plan, further assuring the dosimetric quality of the procedure.

6, In abstract, LDR should be included to specify this is LDR prostate study. It would be better to have clinical suggestions in abstract and conclusion.

Authors’ response: Thank you. We included the term LDR in the abstract, as per your comment. We believe that, with intraoperative optimization, high-quality BT could be achieved with promising clinical outcomes, considering the dose delivered to the prostate [1,2]. We revised our manuscript as follows.

Original: Abstract

This study aimed to evaluate the quality of prostate brachytherapy (BT) based on treatment-related dosimetric outcomes. Data of 100 patients treated using BT with stranded seeds from November 2012 to November 2017 were collected.

Revised:

This study aimed to evaluate the quality of low-dose-rate (LDR) prostate brachytherapy (BT) based on treatment-related dosimetric outcomes. Data of 100 patients treated using LDR BT with stranded seeds from November 2012 to November 2017 were collected.

Original: Page 14, line 296

In our study, compared to the V100% values of 95.72% and 92.23% in the preoperative plan and on day 0, respectively, V100% remained stable on day 30, with a median value of 92.18%.

Revised:

In our study, compared to the V100% values of 95.72% and 92.23% in the preoperative plan and on day 0, respectively, V100% remained stable on day 30, with a median value of 92.18%. Another important dosimetric parameter is D90% for the prostate, as D90% < 130 Gy could be related to an increased risk of recurrence [30]. This parameter was well maintained in our cohort as well, with a median D90% value greater than 150 Gy.

Original: Page 17, line 344

Based on intraoperative dynamic planning using TRUS images, intraoperative in vivo dosimetry using MOSFET, and postoperative dosimetry using the CT images acquired on days 0 and 30, we reported the sustainability of seeds within the desired location, with maintained crucial dosimetric parameters, including the V100% and D90% for the prostate, V100% for the rectum, and V200 Gy for the urethra.

Revised:

Based on intraoperative dynamic planning using TRUS images, intraoperative in vivo dosimetry using MOSFET, and postoperative dosimetry using the CT images acquired on days 0 and 30, we reported the sustainability of seeds within the desired location, with maintained crucial dosimetric parameters, including the V100% and D90% for the prostate, V100% for the rectum, and V200 Gy for the urethra, expecting favorable treatment response.

Sincerely,

Jaeho Cho, MD., Ph.D.

Department of Radiation Oncology, Yonsei University College of Medicine

50-1 Yonsei-ro, Seodaemun-gu, Seoul 03722, Republic of Korea

Tel: +20 82-2-2228-8095; Fax: +82-2-2227-7823

E-mail: jjhmd@yuhs.ac

Reference for revision letter

1.	Morris WJ, Spadinger I, Keyes M, Hamm J, McKenzie M, Pickles T. Whole prostate D90 and V100: a dose-response analysis of 2000 consecutive (125)I monotherapy patients. Brachytherapy. 2014;13(1):32-41. Epub 2013/10/16. doi: 10.1016/j.brachy.2013.08.006. PubMed PMID: 24126102.

2.	Lee WR. Permanent prostate brachytherapy: the significance of postimplant dosimetry. Rev Urol. 2004;6 Suppl 4:S49-56. Epub 2006/09/21. PubMed PMID: 16985870; PubMed Central PMCID: PMCPMC1472865.

3.	Butler, W M et al. “Comparison of seed loading approaches in prostate brachytherapy.” Medical physics vol. 27,2 (2000): 381-92. doi:10.1118/1.598842

4.	Brown, D et al. “Urinary morbidity with a modified peripheral loading technique of transperineal (125)i prostate implantation.” International journal of radiation oncology, biology, physics vol. 47,2 (2000): 353-60. doi:10.1016/s0360-3016(00)00433-8
